# Primary Cardiac Lymphoma: Lessons Learned from a Long Survivor

**DOI:** 10.1155/2016/7164829

**Published:** 2016-12-07

**Authors:** Baljit Singh, Randy Ip, Ahmed Ibrahim Al-Rajjal, Zyad Kafri, Ayad Al-Katib, Tarik Hadid

**Affiliations:** ^1^Graduate Medical Education, St. John Hospital and Medical Center, Detroit, MI, USA; ^2^Graduate Medical Education, St. John Macomb-Oakland Hospital, Detroit, MI, USA; ^3^Van Elslander Cancer Center, Grosse Pointe Woods, MI, USA; ^4^Lymphoma Research Lab., Wayne State University School of Medicine, Detroit, MI, USA

## Abstract

Primary cardiac lymphoma (PCL) is a rare neoplasm that involves the heart, pericardium, or both. Patients with PCL have median survival of approximately 7 months. We report a 63-year-old woman with PCL treated with chemoimmunotherapy but relapsed 7 years later. She received automated implantable cardioverter-defibrillator (AICD) prophylactically shortly after the diagnosis. She presented with a breast recidive 7 years after initial diagnosis and died of relapsed small cell lung cancer. As many patients with PCL die early in the disease course due to life-threatening arrhythmias, preemptive implantation of AICD may improve mortality and prevent early death. Chemoimmunotherapy is effective in inducing remission in patients with PCL. Late and unusual pattern of relapse may be more frequent in patients with PCL and should be explored further. This case presents one of the longest surviving patients with PCL reported in the literature.

## 1. Introduction

Primary cardiac lymphoma (PCL) is a rare type of non-Hodgkin's lymphoma (NHL) that solely involves the heart, the pericardium, or both and accounts for less than 0.01% of all cardiac tumors [1–3]. The clinical presentation of PCL varies depending on the location, size, and degree of invasion [4]. The rising incidence of PCL is thought to be due to advances in diagnostic radiologic technology, greater exposure to environmental toxins, and a larger number of immunocompromised individuals [5]. Here, we present a case of PCL diagnosed intraoperatively and treated successfully with multiagent chemoimmunotherapy with subsequent relapse 7 years after initial diagnosis.

## 2. Case Report

**Figure 1:**
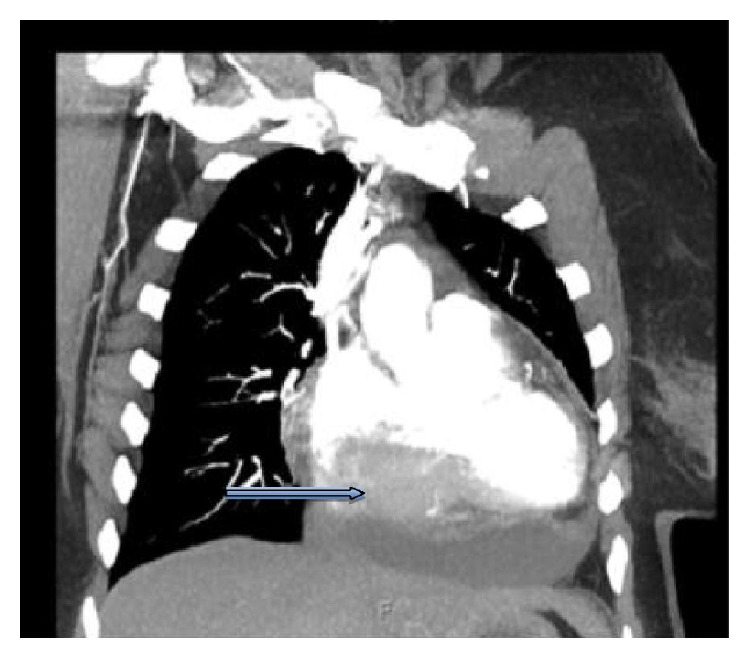
Large right cardiac mass (arrow) on CT angiography.

**Figure 2:**
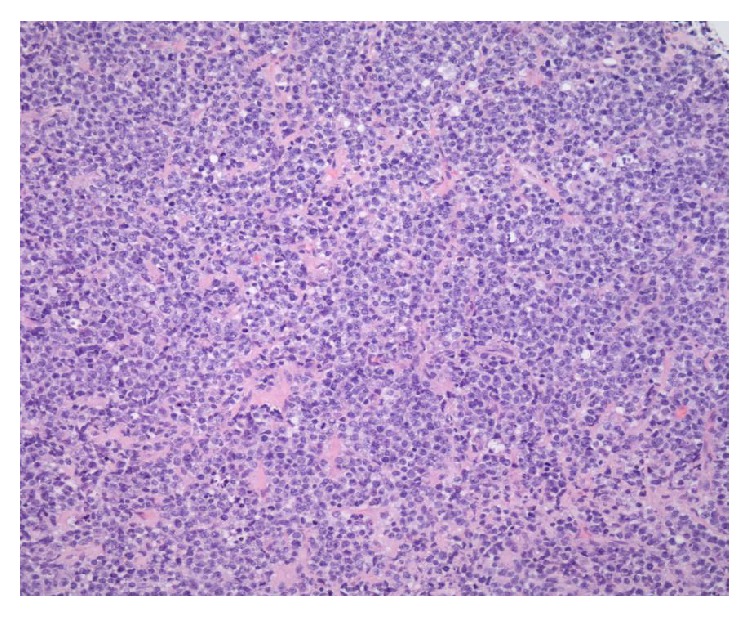
H and E stain of the right atrial biopsy showing sheets of large malignant lymphocytes consistent with DLBCL.

A 63-year-old woman presented with a 3-week history of atypical chest pain, fatigue, and dyspnea on exertion. Physical examination was unremarkable. Electrocardiogram (ECG) showed Mobitz I second-degree atrioventricular (AV) block. A large filling defect in the right atrium and right ventricle was seen on computed tomography (CT) angiography (Figure 1). Transesophageal echocardiogram (TEE) revealed a 56 mm × 48 mm mobile mass arising from the intra-atrial septum, occupying the majority of the right atrial cavity and causing partial obstruction of the right ventricular outflow. Left ventricular (LV) systolic function was preserved with an ejection fraction of 65%. The patient underwent sternotomy with incisional biopsy of the cardiac mass. Pathological examination revealed large malignant lymphocytes expressing CD20, BCL-2, BCL-6, and MUM-1 but not c-myc or cyclin D1 (Figure 2). Ki-67 proliferative index was at 90%. These findings are consistent with diffuse large B-cell lymphoma (DLBCL). CT of the abdomen and pelvis and bone marrow aspiration and biopsy failed to show other areas of disease.

A prophylactic automated implantable cardioverter-defibrillator (AICD) was implanted to prevent sudden cardiac death from cardiac arrhythmias, frequently reported in similar cases [5–8]. She developed a postoperative pulmonary embolism requiring anticoagulation. She achieved complete response (CR) with 6 cycles of chemoimmunotherapy (rituximab, cyclophosphamide, hydroxydaunorubicin, vincristine, and prednisone (R-CHOP)). The patient remained stable for 6 years at which time she was diagnosed with limited-stage small cell lung cancer (SCLC) which was treated with chemoradiotherapy with cisplatin and etoposide resulting in CR and she went on to receive prophylactic cranial irradiation. One year later, she developed a 2 cm solitary right breast mass. Core biopsy revealed DLBCL with an immunophenotype similar to that seen in the original right atrial neoplastic cells except that expression of BCL-6 and MUM-1 was equivocal in the breast biopsy. The Ki-67 proliferative index was at 80%. As LV systolic function was preserved, she was retreated with R-CHOP for 3 cycles achieving CR as confirmed by positron emission tomography (PET)/CT. Within 2 months, she developed a left pleural effusion, suspicious for recurrence of small cell lung cancer. Her condition deteriorated rapidly; she developed multiorgan failure and died 87 months after her initial diagnosis.

## 3. Discussion

PCL is a rare cardiac neoplasm with slight male predominance; median age is 63 at diagnosis [5]. It more frequently affects immunocompromised patients [5, 9, 10]. Involvement of the right side of the heart predominates; only 7% of PCL involves left cardiac chambers [5]. DLBCL is the most common pathological variant of PCL but Burkitt's lymphoma, T-cell lymphomas, small lymphocytic lymphoma, and plasmablastic lymphoma also occur [5, 11]. The most common presenting symptoms are dyspnea followed by constitutional symptoms and chest pain [5]. Approximately 47% of patients with PCL present with congestive heart failure (CHF), which is frequently resistant to standard therapy [12].

With advances in imaging modalities, PCL is being diagnosed more frequently antemortem [8, 11]. The most frequent ECG abnormalities include atrial arrhythmias and AV blocks; however, life-threatening ventricular arrhythmias may occur [5, 8, 13, 14]. CT scans can be helpful in characterizing the extent of cardiac mass [10]. Sensitivity of MRI and TEE at detecting cardiac masses is reported to be greater than 90% but MRI is superior to TEE for detecting myocardial and pericardial thickening [3].

^18^Fluorodeoxyglucose (^18^FDG) PET/CT scanning can be useful in detecting malignant cardiac masses and confirming cardiac isolation [15].

Tissue diagnosis remains of paramount importance. Cytological examination of pleural or pericardial fluid, if present, can detect monoclonal lymphocytes. In a case series of 50 patients, cytological examination of pericardial effusion was diagnostic in 67% of cases [3]. However, some cases still need more invasive diagnostic procedures. Cardiac catheterization with echocardiography-guided transvenous biopsy may be considered when available [13, 16]. Sternotomy with excision of the tumor may be needed to relieve symptoms and obtain tissue for diagnosis [14, 17].

There is no standardized therapy for PCL. Most anecdotal data suggest that since PCL is a systemic disease, therapy should be systemic as well [5]. Multiagent, anthracycline-based chemotherapy is the most widely used therapeutic modality but this can be challenging in patients with LV systolic dysfunction. Rituximab is frequently added due to its established efficacy in NHL [18]. In a small series of patients with PCL, the addition of rituximab to chemotherapy improved progression-free survival from 12 to 24 months [19]. Our patient received chemoimmunotherapy using R-CHOP, which successfully resulted in CR that lasted for 7 years. The role of surgery in PCL treatment is limited. Adding radiotherapy to chemotherapy appears to have limited impact on patient outcomes [5]. The risk of developing radiation-induced heart disease also makes this option less attractive [19, 20]. It is unlikely that adding radiotherapy would have prevented relapse in our patient as it occurred at a distant site.

A few cases of early relapse have been described in the literature [5, 21]. Rarely, isolated extranodal and extracardiac relapse, for example, in the central nervous system, have been reported [21, 22]. Our patient relapsed after 7 years with an isolated breast lesion. The atypical timing and location of relapse we encountered in our patient and reported by others in the literature may suggest a more unique behavior of PCL, one that differs from what is encountered in noncardiac DLBCL, where late relapse is rare with an estimated frequency of 2.2% per year after 2 years of CR [23, 24]. Our patient developed a solid organ neoplasm that may be related to her lifelong tobacco use. However, a link between this diagnosis and PCL and/or its relapse may exist. The use of chemotherapy for small cell lung cancer may have suppressed the immune system allowing for recurrence of previously dormant PCL.

The most common causes of death are intractable heart failure, sepsis, progression of lymphoma, arrhythmias, and sudden cardiac death. Arrhythmias are frequent, occur early in the course of the disease, and may contribute significantly to early death [5, 8, 17, 25]. Reports of AICD use in patients with PCL are scarce and usually follow development of life-threatening arrhythmias [26, 27]. Our patient preemptively received prophylactic AICD, which may explain her long survival of 87 months. The addition of rituximab to the chemotherapeutic regimen may also have contributed to the long survival in our patient. In our opinion, chemoimmunotherapy rather than chemotherapy should be the treatment of choice. Early implantation of prophylactic AICD should be considered in patients with PCL. Finally, clinicians should be vigilant for signs and symptoms of pulmonary embolism and anticoagulation therapy should be promptly initiated as soon as suspicion arises.

## 4. Conclusion

PCL is a rare cardiac neoplasm. Our patient had a late relapse at an extranodal site, suggesting that PCL behavior may be unique and different from that of noncardiac DLBCL. We suggest the use of prophylactic AICD to prevent early deaths due to cardiac arrhythmias, which may be precipitated by the tumor, operative diagnostic procedures, or severe CHF.
